# Prognostic impact of coronary microcirculation abnormalities in systemic sclerosis: a prospective study to evaluate the role of non-invasive tests

**DOI:** 10.1186/ar4136

**Published:** 2013-01-09

**Authors:** Alessandra Vacca, Roberta Montisci, Pietro Garau, Paolo Siotto, Matteo Piga, Alberto Cauli, Massimo Ruscazio, Luigi Meloni, Sabino Iliceto, Alessandro Mathieu

**Affiliations:** 1 University and A.O.U. of Cagliari, Chair and Unit of Rheumatology, S.S. 554 bivio per Sestu, Monserrato 09042, Italy; 2 University and A.O.U. of Cagliari, Chair and Unit of Cardiovascular Diseases, Via Ospedale, Cagliari 09100, Italy; 3 Azienda Ospedaliera Brotzu, Radiology Service, Piazzale Ricchi 1, Cagliari 09121, Italy; 4 University and Azienda Ospedaliera of Padova, via Giustiniani 2, Padova 35128, Italy

## Abstract

**Introduction:**

Microcirculation dysfunction is a typical feature of systemic sclerosis (SSc) and represents the earliest abnormality of primary myocardial involvement. We assessed coronary microcirculation status by combining two functional tests in SSc patients and estimating its impact on disease outcome.

**Methods:**

Forty-one SSc patients, asymptomatic for coronary artery disease, were tested for coronary flow velocity reserve (CFR) by transthoracic-echo-Doppler with adenosine infusion (A-TTE) and for left ventricular wall motion abnormalities (WMA) by dobutamine stress echocardiography (DSE). Myocardial multi-detector computed tomography (MDCT) enabled the presence of epicardial stenosis, which could interfere with the accuracy of the tests, to be excluded. Patient survival rate was assessed over a 6.7- ± 3.5-year follow-up.

**Results:**

Nineteen out of 41 (46%) SSc patients had a reduced CFR (≤2.5) and in 16/41 (39%) a WMA was observed during DSE. Furthermore, 13/41 (32%) patients showed pathological CFR and WMA. An inverse correlation between wall motion score index (WMSI) during DSE and CFR value (r = -0.57, *P *<0.0001) was observed; in addition, CFR was significantly reduced (2.21 ± 0.38) in patients with WMA as compared to those without (2.94 ± 0.60) (*P *<0.0001). In 12 patients with abnormal DSE, MDCT was used to exclude macrovasculopathy. During a 6.7- ± 3.5-year follow-up seven patients with abnormal coronary functional tests died of disease-related causes, compared to only one patient with normal tests.

**Conclusions:**

A-TTE and DSE tests are useful tools to detect non-invasively pre-clinical microcirculation abnormalities in SSc patients; moreover, abnormal CFR and WMA might be related to a worse disease outcome suggesting a prognostic value of these tests, similar to other myocardial diseases.

## Introduction

Until two decades ago, clinical evidence of cardiac involvement in systemic sclerosis (SSc) was considered an infrequent event and it mainly resulted from autopsy studies. In particular, overt manifestations of ischemic heart disease were considered rare, cardiac failure was observed in about 10% of cases, and pericarditis in 15% [1-4]. Conversely, *post mortem *investigations demonstrated myocardial lesions secondary to SSc in more than 50% of cases [2].

The discrepancy between the high prevalence of scleroderma heart involvement (SHI) at autopsy studies and of its lower detection by *in vivo *studies might be due to the low sensitivity or to the scarce applicability of the diagnostic tools utilized [5]. On the other hand, when SHI becomes clinically evident, it assumes a deeply negative prognostic significance, with a mortality rate above 70% at five years [6]. Detailed reviews of the clinical studies concerning SHI have recently been published [7-9]. However, data on the prognostic impact of sub-clinical myocardial involvement as detected by more sensitive tests in SSc patients are presently lacking.

The pathogenesis of SHI is still debated; the most frequent pathological features of SSc in the myocardium are focal fibrosis (in more than 50% of cases) and contraction band necrosis (CBN) (in 77% of patients) [2]. Follansbee *et al. *[10] found a high prevalence of CBN in SSc patients with SHI, probably related to an intermittent vascular spasm of the coronary arteries with episodes of ischemia-reperfusion [2].

The small coronary vessels show a reduced patency or obliteration due to intimal proliferation, fibrinoid necrosis, fibrosis and intravascular coagulation [11]. The microvascular structural and functional abnormalities seem to lead to the increased fibroblast activity and disseminated tissue fibrosis [12], which may progress to a clinical pattern of restrictive cardiomyopathy [13].

The consequences of such anatomical damage and functional disorder were reported in subsequent studies. Kahan *et al. *[14] first demonstrated the impairment of coronary vasodilator reserve using coronary catheterism, later confirmed by non-invasive adenosine transthoracic echocardiography (A-TTE) by other groups [15,16]. In addition, myocardial scintigraphy enabled several authors to observe reversible myocardial perfusion defects, induced either by exposure to the cold or by physical exercise [17-19].

Dobutamine stress echocardiography (DSE) enables evaluation of the dynamics of left ventricular wall motion, which correlate to perfusion and oxygen supply, during chronotropic and inotropic pharmacological stress. This test is a well- established diagnostic and prognostic tool that has widespread applicability because of its clinical accuracy and cost effectiveness [20].

Some authors demonstrated that the simultaneous evaluation of coronary flow velocity reserve (CFR) and left ventricular wall motion (LVWM) by dipyridamole stress echocardiography increases the diagnostic power of each test to detect coronary macro- and micro-vascular involvement [21,22].

In order to increase the accuracy of these two methods, the presence of epicardial artery stenosis should be excluded, and in patients with pre-clinical SHI, where an invasive procedure is not ethically applicable, myocardial multi-detector computed tomography (MDCT) might be preferable in order to avoid cardiac catheterization [23].

On this basis, the presence of early myocardial functional changes in SSc patients asymptomatic for coronary artery disease (CAD) was investigated by combined A-TTE and DSE, integrating them, when applicable, with MDCT, and the impact of such abnormalities on mortality was determined.

## Materials and methods

The initial population comprised 97 SSc patients, who fulfilled the American College of Rheumatology classification criteria [24], followed from September 2000 to June 2006 at our Department. Exclusion criteria were: 1) technically poor acoustic window precluding satisfactory imaging of the left ventricle (for 2-D echo) or of left anterior descending coronary artery (LAD) flow by Color Doppler (for CFR assessment) (16 patients), 2) a right ventricular systolic pressure >40 mmHg by echocardiography (12 patients), 3) hemodynamic instability (2 patients), valvular disease (5 patients), CAD (3 patients), unstable angina (1 patient), life-threatening arrhythmias (1 patient), severe lung and/or pulmonary vascular disease (6 patients), 4) asthma or severe chronic obstructive pulmonary disease (6 patients), and 5) inability or refusal to give informed consent (6 patients). None of them showed symptoms or signs of congestive heart failure, CAD or arrhythmia. Treatment with calcium channel blockers and prostanoids was suspended 48 hours and 4 weeks before functional tests, respectively. All the studied subjects were requested to avoid xanthine-containing food and drinks for ≥24 hours before evaluation. The study was approved by the ethics committee of Cagliari University Hospital (Italy), and written informed consent was obtained from all participants. The final study population consisted of 41 patients; among them, 30 represented the initial cohort who underwent DSE in our previous study [25]. Hematochemical and serological tests, chest X-ray, lung function tests and CT examination, ECG, basal M-mode and two-dimensional echocardiography were carried out on all subjects.

### Follow-up

Following initial cardiac evaluations, all patients were followed up for a mean period of 6.7 ± 3.5 years.

### Adenosine Transthoracic Echochardiography (A-TTE)

In all patients, CFR was assessed by A-TTE as previously described [15]. Briefly, CFR was evaluated in the LAD with transthoracic Color Doppler during adenosine infusion. The pulsed wave Doppler examination of blood flow velocity was recorded in the LAD at rest and after maximum vasodilation by adenosine infusion (140 μg/kg/min for three to five minutes). When the Doppler signal was suboptimal, Levovist^® ^(Schering AG, Berlin, Germany), a suspension of monosaccharide (galactose) microparticles in sterile water, was infused at a concentration of 300 mg/ml, at a rate of 0.5 to 1 ml/min. The CFR value was expressed as the ratio of peak diastolic velocity during hyperaemia to peak diastolic velocity at rest (baseline). CFR values ≤2.5 were considered impaired, as previously reported [15].

### Dobutamine stress echocardiography protocol

All patients underwent DSE. After baseline data had been acquired, dobutamine was infused, beginning at a dose of 5 mcg/Kg/min, and increasing every three minutes to a maximum dose of 40 mcg/Kg/min. Echocardiograms were recorded at baseline, low dose, peak dose and five minutes into recovery. When necessary, atropine (up to 1 mg) was given intravenously at the higher dose levels to augment heart rate response. Assessment was performed by two experienced investigators who had no knowledge of CFR data. The following scoring system was used for regional wall motion: normal = 1; hypokinetic = 2; akinetic = 3; diskinetic = 4. For segmental analysis of left ventriculum (LV) function, a 16-segment model was used as suggested by the American Society of Echocardiography [26]. A Wall Motion Score Index (WMSI) was calculated at baseline and at peak stress as the sum of scores divided by the number of analyzed segments.

End points for dobutamine infusion were: achievement of target heart rate (85% of the maximal heart rate predicted for age), maximal dose of dobutamine and atropine, extensive new wall-motion abnormalities, >2 mm ST-segment depression in two or more ECG leads, chest pain, significant arrhythmias, severe hypertension (blood pressure >220 mmHg) or hypotension (a fall in systolic blood pressure >30 mmHg). ECG and blood pressure were monitored continuously and recorded at each stage.

### Coronary multi-detector computed tomography

The indication to perform MDCT was based on the presence of WMA at the DSE test. MDCT was carried out using a multi-detector computerized tomograph with eight lines of detectors and a rotation time of 500 msec on 360° and with a slice depth of 1.3 mm (Light Speed Ultra, General Electric Medical Systems, Milwaukee, WI, USA). Retrospective gating was used. A total of 120 ml of non-ionic contrast medium was injected at 4 ml/sec. Images were processed as appropriate on a SUN-80 ULTRA workstation (Sun Microsystems, Palo Alto, CA, USA). The entire examination lasted from 20 to 30 minutes. Image evaluation was carried out by an expert radiologist aware of patients' diagnoses.

### Statistical analysis

All data are presented as mean ± SD. Comparisons were made with an independent *t*-test or a non-parametric test (Mann-Whitney, chi-square or Fisher's exact test), when appropriate. Relationships between CFR, WMSI and protocol variables were evaluated with the simple linear correlation coefficient r (determination coefficient r^2^), *P-*values less than or equal to 0.05 were considered significant.

Kaplan-Meier method survival curves were used to summarize the follow-up experience in these patients. The differences in survival curves were tested with a log-rank statistic. The association of selected variables with outcome was assessed with the Cox proportional hazard model using univariate and stepwise multivariate procedures. A significance of 0.05 was required for a variable to be included in the multivariate model, whereas 0.1 was the exclusion cut-off value. Hazard ratios with the corresponding 95% confidence intervals were estimated. Data management and analysis were performed using MedCalc software (version. 12.2.1; Mariakerker, Belgium).

## Results

Forty-one consecutive SSc patients (33 female, 8 male; mean age 54.1 y, range 28 to 73) with mean disease duration of 7.5 y (range 1 to 22 y) were included in the study. Fifteen out of the 41 SSc patients were affected by dcSSc subtype and 26 by lcSSc form, according to the LeRoy *et al*. classification [27]. Demographic, clinical and laboratory data of patients are reported in Table 1.

**Table 1:** Demographic and clinical features of systemic sclerosis patients

	dcSSc		lcSSc		
***Demographic and clinical features***	**Data**	**Range**	**Data**	**range**	***P-*value**
**Number of patients**	15		26		
**Age (yrs; mean; SD)**	53 ± 13.1	28 to 71	52 ± 9.3	39 to 73	NS
**M/F ratio**	2/13		6/20		NS
**Disease duration (yrs; mean; SD)**	8 ± 3.3	2 to 15	8.5 ± 5.8	1 to 21	NS
***Clinical manifestation***					
**Raynaud phoenomenon**	15 (100%)		23 (88%)		NS
**Teleangectasias**	10 (66%)		9 (35%)		NS
**Lung involvement**	13 (87%)		14 (54%)		NS
**Oesophagus involvement**	14 (93%)		9 (35%)		0.001
**Trophic ulcers**	10 (66%)		8 (31%)		0.06
**Calcinosis**	0		5 (19%)		NS
***Serological findings ***					
**Anti-Scl-70**	12 (80%)		10 (38%)		0.02
**Anti-Centromere**	0		6 (23%)		NS
***Risk factor for CAD***					
**Hypertension**	4 (27%)		9 (35%)		NS
**Diabetes**	2 (13%)		2 (7%)		NS
**Hypercholesterolemia**	6 (40%)		4 (15%)		NS
***Treatment***					
**D-Penicillamine**	3 (19%)		5 (19%)		NS
**Cyclophosphamide**	8 (50%)		0		0.0004
**Cyclosporine**	3 (19%)		3 (11%)		NS
**Iloprost**	13 (87%)		9 (35%)		0.003
**Steroids**	6 (37%)		2 (7%)		0.004
**Calcium-blockers**	3 (19%)		1 (3%)		NS
**ACE-inhibitors**	3 (19%)		1 (3%)		NS
**Angiotensin II receptor blocker**	2 (13%)		1 (3%)		NS

Echocardiographic and functional cardiac parameters determined in SSc patients studied are listed in Tables 2 and 3. Both cardiac stress tests were well tolerated and no cardiac adverse events occurred during examinations. The median interval time between A-TTE and DSE was 1.5 weeks.

**Table 2:** Echocardiographic and hemodynamic parameters of systemic sclerosis patients

Cardiac parameters	Mean ± SD
LV diastolic diameter (mm)	44.9 ± 6.3
LV EF (Simpson Biplano) (%)	66.0 ± 5.4
Thickness of the interventricular septum (mm)	9.5 ± 1.3
E/A	1.0 ± 0.3

E/A, early/late diastolic velocity ratio; EF, ejection fraction; LV, left ventricle

**Table 3:** Cardiac parameters during dobutamine stress echocardiography (mean values ± standard deviation)

	Pts with normal DSE (*n *= 25)	Pts with abnormal DSE (*n *= 16)	*P-*value
***Heart rate(beats/min*)**			
Baseline	75.3 ± 17.3	79.3 ± 8	ns
Max DSE	138 ± 12.5	129.7 ± 17.2	ns
***Sistolic BP (mmHg)***			
Baseline	120 ± 18.7	117.5 ± 14.4	ns
Max DSE	136.1 ± 22.9	131.6 ± 20.3	ns

BP, blood pressure; DSE, dobutamine stress echocardiography test; Max, maximal; ns, not significant

**Figure 1:**
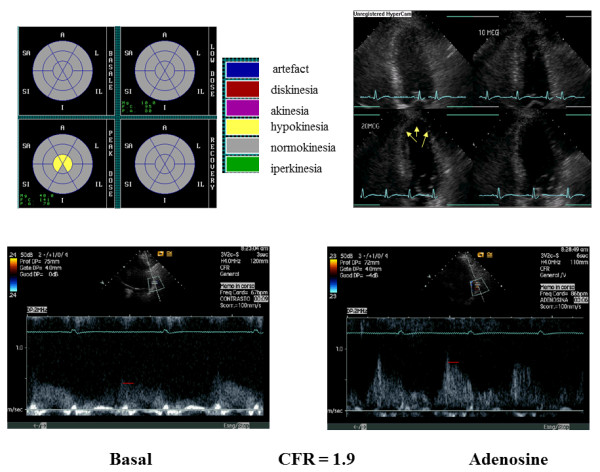
**Doppler echocardiography image of a SSc patient with WMA and impaired CFR**. Doppler echocardiography image of a patient with wall motion abnormalities (hypokinesia of apical segments) during Dobutamine Stress Echocardiography (upper panel) and abnormal Coronary Flow Reserve (lower pannel).

Nineteen out of 41 (46%) patients with SSc showed reduced CFR (≤2.5) and in 16/41 (39%) SSc patients WMA (hypokinesia), which was absent on baseline rest examination, was observed during dobutamine infusion (Figure 1). Thirteen out of 41 patients (32%) showed both CFR and DSE test impaired; these combined abnormal findings were more frequent in the subgroup with dcSSc (8/15; 53%) than in the lcSSc subset (5/26; 19%) (*P *<0.04), while they were irrespective of age, disease duration, presence of anti Scl-70 or anti-centromere antibody, esophageal and lung involvement, digital ulcers, high cholesterol serum levels and blood pressure.

WMA during DSE were limited to one segment in 10 patients, to two segments in 5 patients and to three segments in 1 patient. In seven patients with abnormal DSE, WMA were localized in the LAD coronary artery area, in three patients in both the LAD and right coronary artery area, whereas in three patients wall motion abnormalities were only in the right coronary artery area and in three patients in circumflex coronary area.

**Figure 2:**
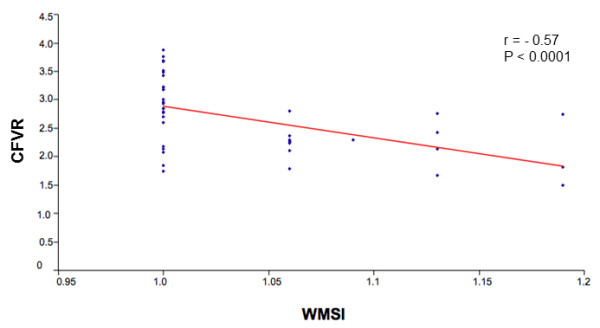
**Inverse correlation between CFR and WMSI in examined SSc patients**. Relationship between coronary flow reserve (CFR) and wall motion score index (WMSI) in systemic sclerosis patients. WMSI = difference between rest and peak WMSI (0 to 1 minute after the end of peak dose).

An inverse correlation between CFR and WMSI (r = -0.57, *P *<0.0001) was observed (Figure 2). Moreover, CFR was significantly reduced in patients with induced WMA during DSE (2.21 ± 0.38 vs 2.94 ± 0.60, *P *= <0.0001); in detail, 13/16 patients (81%) with WMA during DSE also had impaired CFR and only 3 had normal CFR, while among patients without WMA only 6 (24%) showed pathologically reduced CFR and 19 had normal values (*P *<0.0001).

The presence of epicardial artery stenosis, which might affect the two functional tests' results, could be excluded by MDCT in 12 patients with WMA, as the examination was declined by 3 patients and 1 died before MDCT was performed.

### Survival analysis

Following cardiac examination, patients were followed for 6.7 ± 3.5 years and 9/41 patients (20%) died. Death was related to lung malignancy in two patients and to progression of scleroderma in seven (renal crisis in one patient, sudden death in three, pulmonary arterial hypertension in two and acute respiratory failure in one).

It is worth noting that all seven patients who died as a result of disease progression had impaired CFR and six had WMA at the beginning of follow-up; moreover, they all had the dcSSc form. In summary, six deaths occurred among the 13 patients with combined pathological tests for coronary microvascular function compared to one death among the remaining 28 without occurrence of combined abnormalities at the functional tests (*P *<0.01).

**Figure 3:**
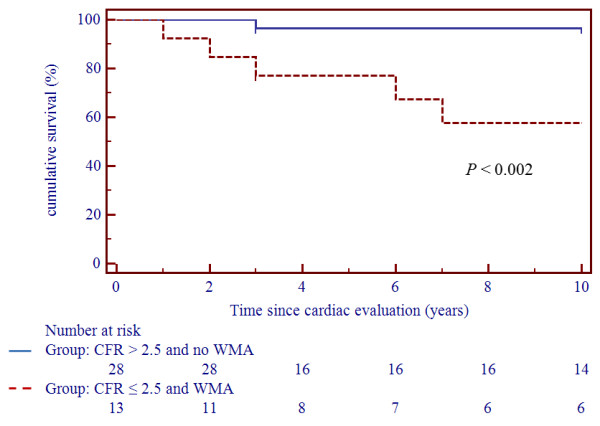
**Kaplan-Meier survival curves in patients stratified according to normal CFR/no WMA and abnormal CFR/WMA**. Kaplan-Meier survival curves in patients stratified according to normal coronary flow reserve (CFR >2.5) and no wall motion abnormalities (WMA) versus abnormal CFR (CFR ≤2.5) and WMA at Doppler echocardiography. The worst survival is observed in patients with abnormal CFR.

Univariate analysis showed that dcSSc (*P *= 0.01, HR 8.17, 95% CI 1.82 to 36.63), impaired CFR (*P *= 0.006), WMA (*P *= 0.02, HR 8.05, 95% CI 1.80 to 36.02), and the combination of CFR impairment and WMA (*P *= 0.002, HR 12.74, 95% CI 2.60 to 62.36) were predictors of unfavorable prognosis. Multivariate analysis confirmed that simultaneous CFR impairment and WMA were independent risk factors for mortality (*P *= 0.003). Kaplan-Meier analysis showed 10-year survival rates of 96.5% and 59%, respectively, in patients with simultaneous CFR >2.5 and no WMA versus those with CFR ≤2.5 and WMA (*P *<0.002) (Figure 3).

## Discussion

Early detection of SHI is related to the sensitivity of the diagnostic tools used. The different methods reported in available literature evaluated either functional disorders of coronary microcirculation or functional myocardial abnormalities. The first include evaluation of CFR with invasive and non-invasive tests [14,15] and evaluation of myocardial perfusion with SPECT and MRI [17,18,28-30]. The second include evaluation of systolic or diastolic dysfunction by Doppler echocardiography [31-35] and tissue-Doppler echocardiography [36].

To our knowledge, this is the first report where two non-invasive methods, one exploring coronary microvascular function (A-TTE test) and the other perfusion- and metabolism-dependent contractility (DSE test), have been combined and complementarily applied in SSc to detect pre-clinical cardiac involvement; furthermore, we suggested that CFR and DSE impairment might have an impact on the prognosis of such patients.

Our findings can be summarized as follows. First, impaired myocardial microcirculation has been confirmed in SSc patients in the absence of any CAD related symptoms and signs as revealed by non-invasive methods whose diagnostic concordance is good [21,22]. Second, the combined CFR impairment and WMA occurrence seem to identify a subset of SSc patients at higher risk for a worse outcome and might provide the potential to closely monitor and counteract myocardial damage with more aggressive treatments, whereas patients with normal CFR and negative stress tests have showed a favourable outcome (no deaths in this group during follow-up).

Our study demonstrated the complementary use of CFR and WMA in SSc. CFR impairment, already reported in a smaller series of SSc patients [15], in the absence of major epicardial coronary arteries stenosis [23], could explain the segmental kinetic abnormalities during pharmacologic stimulus. In fact, the genesis of the LVWMA induced by dobutamine infusion is likely to be linked to the relative ischemia secondary to inotropic and chronotropic stimuli in patients with impaired CFR. As already demonstrated in other series of patients at high risk of CAD, the combined result of low CFR and abnormal LVWM should address stenosis of epicardial arteries [22], but as is reported in the literature, macrovascular disease is considered in SSc as frequent as expected in the general population [37-39]. An inverse correlation between WMSI and CFR was found in our SSc patients, similar to other pathological conditions where microvascular dysfunction is present, such as after reperfusion in acute myocardial infarction [40] and dilated cardiomyopathy [41]. During dobutamine infusion, an increase in oxygen consumption occurs; thus if concomitant microvascular dysfunction with reduced CFR is present, we can hypothesize that the mismatch between oxygen demand and supply could induce regional LVWMA. On the other hand, the incomplete overlap between patients with abnormal DSE and with impaired CFR may be explained by the fact that CFR is a more sensitive parameter of microvascular impairment and could be an early marker of endothelial dysfunction, which does not normally induce regional WMA.

Another interesting finding in our series is the absence of WMA abnormalities at baseline examination, suggesting that they are neither directly referable to the patchy areas of fibrosis reported in the literature [2,42] nor to atherosclerotic CAD, but rather to the expression of a functional defect, preceding the anatomical lesion, as seen during stress [25]. Moreover, WMA did not correspond to any epicardial coronary artery distribution, as demonstrated by cardiac MDCT; therefore, they are highly suggestive of microvascular abnormalities [23,39].

The CFR impairment and DSE pattern abnormalities observed in our SSc patients seem to be a direct expression of SHI, and it is conceivable that these alterations might have an unfavorable impact on the disease outcome.

In the current study, we found that the presence of abnormal CFR and/or WMA was related to a worse outcome. In fact, the high mortality observed in the group with both tests abnormal suggests that coronary microcirculation dysfunction, although clinically silent, needs a more specific and earlier therapeutic approach. Although only three of the patients with abnormal myocardial tests died of cardiac related causes, we cannot exclude that impaired CFR and WMA could be one of the early expressions of microcirculation involvement which might influence prognosis, independently of the cause of death. In this context, the evidence of microcirculation abnormalities may be a manifestation of present or future severe heart disease. It has been described that reduced CFR, in the absence of CAD, negatively influences prognosis in different myocardial diseases [41,43,44]; looking at these studies, CFR might become a new therapeutic target in the near future [45].

Our data also highlighted a significantly elevated frequency of both CFR and WMA in patients with dcSSc in comparison to those with lcSSc. Since the mean disease duration appears to be similar in patients with the two cutaneous disease subsets, this difference might be the expression of a more rapid and/or severe progression of myocardial involvement in dsSSc, in agreement with the different timing of development of visceral lesions in the two SSc subsets.

Evaluation of coronary microcirculation with A-TTE and DSE appears to be complementary to the standard echo-color-Doppler technique currently utilized in routine assessment of cardiac abnormalities detectable in SSc patients (pulmonary hypertension, ventricular hypertrophy, valve lesions, alterations of diastolic or systolic function); moreover, when compared to other stress imaging techniques with comparable prognostic value, such as myocardial perfusion scintigraphy, stress echocardiography has clear advantages: lower cost, higher specificity (no need for coronary angiography) and lack of radiation exposure. Doppler echocardiography CFR may be a suitable tool in this clinical condition, while the potential role of CT scanning has yet to be established, and its costs and limited availability do not allow its routine use in clinical practice [43,44,46].

Our data have some limitations as this study was carried out in a series of patients from a single clinical center; therefore, further studies by other investigators in a larger cohort are required to confirm our results; the examinations are dependent on image quality and need an expert reader. The high radiation exposure with MDCT although lower than catheterization, is another aspect which should be taken into account [47]. The relatively small number of deaths can reduce the statistical power on the mortality data.

Further studies should also target the potential use of A-TTE and DSE in evaluating the benefits of pharmacological treatment on small coronary vessel disease in SSc. An example of that is the recent evaluation of the effects of a non-conventional short-term treatment (l-propionylcarnitine) on the coronary microvasculature in SSc patients with impaired CFR [48].

## Conclusions

In summary, we confirmed pre-clinical coronary microcirculation impairment in a large series of SSc patients, asymptomatic for CAD, by two complementary non-invasive tests. During a 10-year follow-up, we observed that patients with coronary microcirculation abnormalities had a worse prognosis.

An extension of the follow-up of the patients evaluated in this study is in progress in order to further assess the power of A-TTE and DSE in providing an early prospective index of silent SHI progression and a reliable prognostic score in SSc.

## Abbreviations

A-TTE: transthoracic-echo-Doppler with adenosine infusion; CAD: coronary artery disease; CBN: contraction band necrosis; CFR: coronary flow velocity reserve; DSE: dobutamine stress echocardiography; LAD: left anterior descending coronary artery; LVWM: left ventricular wall motion; MDCT: multi-detector computed tomography; SHI: scleroderma heart involvement; SSc: systemic sclerosis; WMA: wall motion abnormalities; WMSI: wall motion score index

## Competing interests

The authors declare that they have no competing interests.

## Authors' contributions

AM, SI and LM conceived of the study and participated in its design and coordination. AV and PG participated in the design of the study, enrolled the patients and wrote the manuscript. RM performed cardiologic examinations, helped to draft the manuscript and performed the statistical analysis. PS performed MDCT examination. MP, AC and MR helped to perform the statistical analysis and draft the manuscript. All authors read and approved the final manuscript.
